# Solidarity and reciprocity during the COVID-19 pandemic: a longitudinal qualitative interview study from Germany

**DOI:** 10.1186/s12889-023-17521-7

**Published:** 2024-01-02

**Authors:** Franziska B. Schönweitz, Bettina M. Zimmermann, Nora Hangel, Amelia Fiske, Stuart McLennan, Anna Sierawska, Alena Buyx

**Affiliations:** 1 Institute of History and Ethics in Medicine, Department of Clinical Medicine, TUM School of Medicine and Health, TUM School of Social Sciences and Technology, Technical University of Munich, Munich, Germany; 2 Institute of Philosophy and Multidisciplinary Center for Infectious Diseases, University of Bern, Bern, Switzerland; 3 Leibniz Center for Science and Society (LCSS), Leibniz University of Hannover, Hannover, Germany; 4 Institute for History of Medicine, Technical University of Dresden, Dresden, Germany

**Keywords:** Social cohesion, Ethics, SARS-Coronavirus-2, Solidarity, Reciprocity

## Abstract

**Background:**

While solidarity practices were important in mitigating the Coronavirus Disease 2019 (COVID-19) pandemic, their limits became evident as the pandemic progressed. Taking a longitudinal approach, this study analyses German residents’ changing perceptions of solidarity practices during the COVID-19 pandemic and examines potential reasons for these changes.

**Methods:**

Adults living in Germany were interviewed in April 2020 (n = 46), October 2020 (n = 43) and October 2021 (n = 40) as part of the SolPan Research Commons, a large-scale, international, qualitative, longitudinal study uniquely situated in a major global public health crisis. Interviews were analysed using qualitative content analysis.

**Results:**

While solidarity practices were prominently discussed and positively evaluated in April 2020, this initial enthusiasm waned in October 2020 and October 2021. Yet, participants still perceived solidarity as important for managing the pandemic and called for institutionalized forms of solidarity in October 2020 and October 2021. Reasons for these changing perceptions of solidarity included (i) increasing personal and societal costs to act in solidarity, (ii) COVID-19 policies hindering solidarity practices, and (iii) a perceived lack of reciprocity as participants felt that solidarity practices from the state were not matching their individual efforts.

**Conclusions:**

Maintaining solidarity contributes to maximizing public health during a pandemic. Institutionalized forms of solidarity to support those most in need contribute to perceived reciprocity among individuals, which might increase their motivation to act in solidarity. Thus, rather than calling for individual solidarity during times of crisis, authorities should consider implementing sustaining solidarity-based social support systems that go beyond immediate crisis management.

**Supplementary Information:**

The online version contains supplementary material available at 10.1186/s12889-023-17521-7.

## Introduction

The Coronavirus Disease 2019 (COVID-19) pandemic and its profound implications for societies around the globe brought the importance of solidarity as a lived social practice to the forefront of public policy and debate. Particularly in the initial stages of the COVID-19 pandemic, solidarity was invoked by policymakers to alert people to the necessity of their collective help in addressing the crisis [1–3]. The most prevalent normative argumentation to justify lockdowns and other severe restrictions on personal freedom was to protect elderly or vulnerable people, healthcare professionals, and the healthcare system as a whole. This was captured in calls to “flatten the curve,” where acting together was portrayed as slowing down the spread of the virus and eventually saving lives [4, 5]. Indeed, solidarity practices and other pro-social behaviours are important motivators for compliance with protective policy measures during the COVID-19 pandemic [6].

Solidarity practices are primarily manifested through various forms of support for those deemed vulnerable in a particular situation and involve people caring and taking responsibility for each other [7, 8]. Through acting in solidarity, a sense of belonging and togetherness is established. Following Prainsack and Buyx [9], we understand solidarity as “an enacted commitment to carry ‘costs’ (financial, social, emotional or otherwise) to assist others with whom a person or persons recognise similarity in a relevant respect”. What is considered “similarity in a relevant respect” is determined by the specific situations within which solidarity takes place. Moreover, solidarity can manifest itself at an interpersonal level, a group level, and at the level of formal institutions and norms (institutionalized solidarity) [9, 10]. Institutionalized solidarity involves indirect reciprocity by redistributing costs and benefits of solidarity practices, i.e. through policies intended to support particularly vulnerable groups in society, or legal rules to help enforce health-related policies or equitable sharing of resources. For example, affordable public health care or taxation according to income is built on this sort of reciprocal institutionalized solidarity [11].

In sum, we understand solidarity as a *practice* that (i) comes with *costs*, excluding sentiments and purely ideological support; (ii) is motivated by context-relevant *similarities* [9, 12] (distinguishing it from charity); (iii) is stabilized by some form of perceived *reciprocity*, particularly at the institutional level [9] (distinguishing it from altruism); and (iv) is to be distinguished from friendship and love, which are deeper feelings that render solidarity redundant [9].

In the early stages of the COVID-19 pandemic, researchers emphasized the importance of solidarity to overcome the pandemic crisis while warning about the potential of polarization [7, 13]. Empirical studies reported widespread solidarity practices in the early stages of the pandemic such as increased hygiene, social distancing, taking care of vulnerable groups, or supporting healthcare professionals [8, 14]. It was also shown that fostering solidarity and the resulting social cohesion improved crisis preparedness [15, 16]. Solidarity was further confirmed to be one of several motivators for complying with restrictive policies in the context of COVID-19 [6, 17]. Further studies focused on solidarity as a motivation to wear face masks [18, 19] or get vaccinated against COVID-19 [20, 21]. Solidarity was also discussed in the context of the worldwide distribution of COVID-19 vaccines [22–24].

At the same time, however, the limits in practising solidarity became apparent as the pandemic progressed. Studies focusing on intergenerational solidarity, for example, stressed the problem of ageist stereotyping (people are patronized, excluded, pitied, or blamed because of their age) caused by the narrative of protecting elderly people in public and political discourses [25–27]. Others shed light on the failure of a fair worldwide COVID-19 vaccine distribution [28, 29]. Further studies emphasized that the COVID-19 pandemic made the limits of solidarity visible on an institutional level. For example, Flynn (2022) concluded that in Ireland, “underfunded public system[s] have eroded solidarity weakening its effectiveness” and that there was an acute need to invest more in collective social capital [30].

When discussing what could motivate solidarity practices on all levels, reciprocity was a frequently mentioned prerequisite. For example, in the 2020 issue of the European Governance & Politics Programme of the European University Institute, Cicchi et al. (2020) concluded that “in a large heterogeneous union, reciprocity is a far sounder basis for solidarity than moral or identity” [31, 32]. Another survey-based study from Switzerland found reciprocity as a motivating factor for solidarity practices on the individual and group levels during the COVID-19 pandemic [33].

Solidarity and reciprocity have also been extensively discussed and investigated in the context of other international crises, such as the migration crisis in Europe [34] or climate change [35]. These discussions show the importance of fostering solidarity on the institutional and political levels as well as the interpersonal level. To meet such a global crisis, solidarity must be inherently connected with improving social justice and equity on a global level [36].

The COVID-19 pandemic represents a recent, multi-faceted, global health crisis where solidarity practices were seen as crucially important for successful management. Identifying the limits of solidarity practices contributes to the acknowledgement and reinforcement of these practices, which are particularly important during a crisis. However, there are few empirical studies assessing the progression and limits of solidarity practices during a long-lasting crisis, such as the COVID-19 pandemic. Thus, drawing from a unique set of longitudinal qualitative interviews held during the COVID-19 pandemic, this study aims to investigate German residents’ changing perceptions of solidarity practices during this pandemic and examine potential reasons for these developments. This study expands a previous analysis based on interviews held in April 2020, where we reported a high sense of togetherness and consideration for elderly people despite acknowledged limits of solidarity practices [14]. Based on these empirical findings, we develop suggestions for how solidarity practices during crisis can be fostered, maintained, and promoted.

## Methods

This study has been made possible by the joint work of the members of the “Solidarity in Times of a Pandemic” (SolPan) Research Commons. SolPan is a qualitative, longitudinal, multinational study conducted during the COVID-19 pandemic to gain insights into people’s lived experiences and viewpoints to understand how and why people engage in solidarity practices or not [37]. SolPan included ten European countries and a previous publication reported international findings from April and October 2020 [38]. This study, by contrast, focuses on Germany and builds on a previous study by our group that focused on the initial stages of the pandemic (interviews held in April 2020, T1, n = 46, see [14]). Thus, we analyze all interviews held with German residents in October 2020 (T2, n = 43) and October 2021 (T3, n = 40), occasionally referring to insights from T1. This allows for a more in-depth focus on the longitudinal progression of people’s lived experiences and viewpoints as the pandemic progressed. The Technical University of Munich’s ethics committee approved the study (no. 208/20 S). We applied the COREQ checklist for qualitative research reporting [39].

Most co-authors conducted interviews in Germany (FS (M.Sc., female), BZ (Dr., female), NH (Dr., female), AF (Dr., female), AS (M.A., M.Sc., M.Phil., female), AB (Prof. Dr., female)) except for SM (Dr., male) who conducted interviews in other SolPan countries. All co-authors were employed as researchers at the time of the interview and were formally trained in qualitative interview techniques. Except for FS, all co-authors had prior experience in qualitative research and interviewing. Because of resource constraints and the ad-hoc set-up of this study, some participants were interviewed by the same researcher every time, whereas others spoke with different researchers. All interviewers were instructed to familiarize themselves with the transcripts of previous interviews to be able to prompt follow-ups or changes in participants’ perceptions.

### Recruitment

Because of the ad-hoc setup of the project in light of the sudden advent of the COVID-19 pandemic and its fast-changing nature, the SolPan Research Commons applied a pragmatic recruitment strategy to collect interviews in a short period [40]: Participants were initially recruited in March and April 2020 through online advertisement on university websites, social media, snowballing and convenient sampling and were re-contacted for subsequent interviews. Adult, legally competent residents of Germany were eligible to participate.

Because of the rapid evolution of the COVID-19 pandemic, we considered it important to interview all participants within a limited time frame (approx. 5 weeks per round). We also wanted to portray a cross-sectional, qualitative picture of people’s perceptions rather than focusing on one particular group. This approach had the disadvantage that theoretical saturation could only be assessed post hoc (see limitations). We, therefore, followed a pragmatic approach to theoretical saturation as proposed by Jacqueline Low [41], who suggested assessing theoretical saturation through conceptual rigour, aiming for robustness and coherence during data analysis [41].

To facilitate a variety of perspectives, in recruitment a range of different demographics, including age, gender, income, household structure, geographic area, education, and employment were controlled for (Table 1). Participants were contacted via email or telephone. In October 2020, three out of 46 participants dropped out of the study and overall six in October 2021. They did not respond to the interview invite or stated time constraints as a reason for not wanting to participate anymore.

**Table 1:** Demographic distribution of participants in Germany

Category	T1	T2	T3
**Age**			
18–30	9 (19.6%)	7 (16.3%)	8 (20.0%)
31–45	19 (41.3%)	18 (41.9%)	16 (40.0%)
46–60	5 (10.9%)	5 (11.6%)	4 (10.0%)
61–70	8 (17.4%)	8 (18.6%)	8 (20.0%)
70+	5 (10.9%)	5 (11.6%)	4 (10.0%)
**Gender**			
Female	24 (52.2%)	23 (53.5%)	21 (52.3%)
Male	22 (47.8%)	20 (46.5%)	19 (47.5%)
Other	0 (0%)	0 (0%)	0 (0%)
**Household**			
Single	13 (28.3%)	13 (30.2%)	13 (32.5%))
Couple	16 (34.8%)	15 (34.9%)	14 (35.0%)
Living with child/children under 12	8 (17.4%)	7 (16.3%)	3 (7.5%)
Living with child/children 12+	4 (8.7%)	4 (9.3%)	6 (15.0%)
other	5 (10.9%)	4 (9.3%)	4 (10.0%)
**Geographic Location**			
Big town (e.g. capital, + 500k)	22 (47.8%)	22 (51.2%)	22 (55.0%)
Medium/small town	12 (26.1%)	11 (25.6%)	8 (20.0%)
Rural (e.g. village)	12 (26.1%)	10 (23.2%)	10 (25.0%)
**Employment status**			
Employed (long-term)	21 (45.7%)	21 (48.8%)	20 (50.0%)
Self-employed	4 (8.7%)	4 (9.3%)	4 (10.0%)
Employed (short-term/precarious)	3 (6.5%)	3 (7.0%)	2 (5.0%)
Unemployed	4 (8.7%)	3 (7.0%)	2 (5.0%)
Retired	10 (21.7%)	10 (23.2%)	10 (25.0%)
other	4 (8.7%)	2 (4.7%)	2 (5.0%)
**Education level**			
Less than 10 years	2 (4.3%)	2 (4.6%)	2 (5.0%)
10–14 years (e.g. high school diploma)	16 (34.8%)	14 (32.6%)	12 (30.0%)
Higher education	28 (60.9%)	27 (62.8%)	26 (65.0%)
**Household net income**			
Up to 1,400€/month	5 (10.9%)	2 (4.6%)	4 (10.0%)
1,401-3,000€/month	14 (30.4%)	15 (34.9%)	10 (25.0%)
More than 3,000€/month	27 (58.7%)	26 (60.5%)	26 (65.0%)
**Total**	**46 (100%)**	**43 (100%)**	**40 (100%)**

### Data collection

Before the interviews, participants received written information and an oral explanation from the interviewer about the study. The interviews were held either via telephone or video calls (e.g., zoom, teams). Only the interviewer and the participant were present at the interview. Before starting the first interview, the interviewers introduced themselves to the participants. Moreover, they briefly explained the goal of the study. After that, participants gave their consent to participate orally; the consent was recorded. The semi-structured interviews lasted 30–100 min and were conducted in German (except for one interview held in English). Interviews were audio recorded and pseudonymized upon transcription. Interview transcripts were not returned to participants.

The interview guides were developed jointly by the SolPan Research Commons, pre-tested and adapted in each interview phase, considering the most recent pandemic developments [42]. In the first two rounds of interviews, we purposefully avoided using the term “solidarity” when asking questions to mitigate socially accepted answers and bias due to the diffuse use of the concept in everyday language. Only at the end of the last interview, participants were asked what solidarity meant to them and were invited to reflect on perceived solidaristic as well as unsolidaristic practices during the COVID-19 pandemic.

### Data analysis

All interviews were coded with Atlas.ti software using a coding scheme that was inductively generated by the SolPan Research Commons [40]. After each series of interviews, pilots were conducted using the coding scheme. If new themes emerged consistently across countries, these were agreed upon in an overarching method workshop and added as additional codes. A second researcher checked the coding for consistency. Overall, four researchers and six research assistants coded the data.

Text passages relevant to solidarity and supporting practices were extracted via the Atlas.ti query function and first analysed inductively and manually (without Atlas.ti). All co-authors were part of the qualitative data analysis process, which proceeded as follows: First, FS and NH identified different aspects of solidarity in the data based on the codes from the SolPan coding scheme. All authors then analysed these aspects separately and wrote analytic reports, based on which FS drafted a summary. NH and BZ then independently reviewed and restructured this summary. Preliminary findings were presented to the authors’ research group and discussed in several meetings. At that point, findings were contrasted against the theoretical lens of solidarity (as outlined in the introduction). Finally, in an iterative process, FS and BZ – with feedback from the other authors – finalized the reporting of the results. Through this process, we aimed at consistency between the data presented, the theoretical lens of solidarity, and the findings. Participants did not provide feedback on the findings.

## Results

**Fig. 1:**
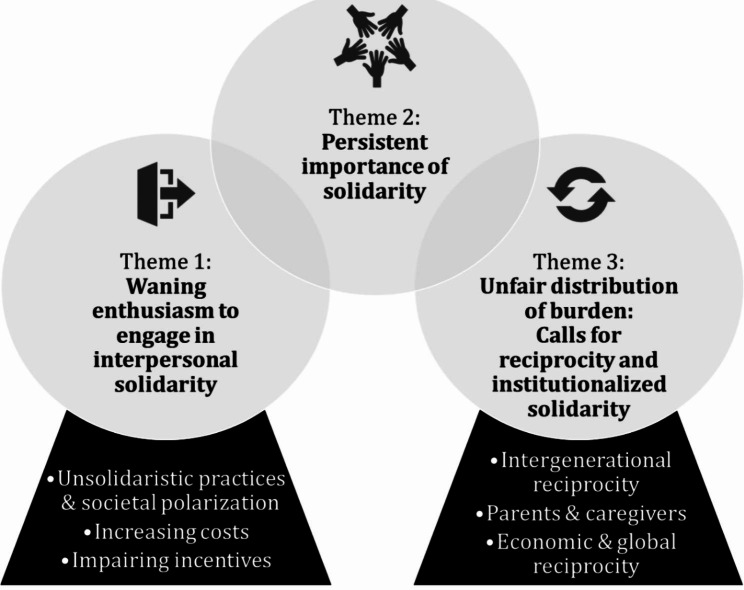
Overview of results

In analysing participants’ changing perceptions of solidarity practices through the theoretical lens in the later stages of the COVID-19 pandemic, we identified three major themes (see Fig. 1 for an overview). First, we present participants’ perceptions of a waning enthusiasm to engage in interpersonal solidarity and three potential reasons (based on data and theory). Second, we report on the persistent importance of solidarity practices to navigate the COVID-19 crisis. Third, we describe participants’ perceptions of an unfair distribution of burden and show how these link to calls for more reciprocity and institutionalized solidarity.

### Waning enthusiasm to engage in interpersonal solidarity

In April 2020, most participants described a strong sense of standing together in solidarity to avoid major harm due to COVID-19. This was also the case for an elderly woman (over 75 years) who stated:

*We used to have a note in our mailbox with phone numbers where you could call and ask them to buy something for you. But we also have young families in our house who have already offered to buy things for us. But I’ve also been active myself, I bring an elderly woman, maybe in her mid-80s, I’m [75+] years old, food twice a week that I’ve cooked myself. It makes very little difference if I cook for two or three people. And it’s just two houses away, right here on our street. And I can give it to her from outside through the kitchen window. She lives upstairs. I’ve been doing this for about three weeks now. (participant 6, April 2020, see also supplementary quotes #1–5)*

By October 2020, however, many participants described how such general motivations began to wane, as this long-term employed mother of young children described:

*Yes, it was very difficult for us personally when it all started. But at the same time, we had the feeling that we were all doing it together. Somehow, we’ll get through it as a society, with lots of initiatives. And now I see a bit of the opposite, especially for us as a family with secure jobs and partly regular processes, a lot has become easier. But at the same time, I also have the feeling that this initial enthusiasm, that we are all in the same boat, is no longer there, for various reasons… On the one hand, many people are exhausted. On the other hand, they don’t know what to believe and what not to believe. (participant 42, October 2020, see also supplementary quotes #6–11)*

When asked about support initiatives in their neighbourhoods, most participants who reported on such initiatives in April 2020 said that they had vanished by October 2020 (even though there were few exceptions, see supplementary quotes #20 + 21). This was not only because those supporting others were less willing to do so, but also because those who had previously been on the receiving end of support had less need for it. For example, this female in her 60s with a high school diploma and a stable job who lived alone described:

*Yes, well, it has eased up now, but because the older people are going out themselves again. Not in the old people’s home, someone still does the shopping for my father, but my former bosses, for example, who I used to do the shopping for, now go themselves, so they don’t have to worry so much. (participant 46, October 2020, see also supplementary quotes #12–19)*

#### Unsolidaristic practices and societal polarization

Alongside waning enthusiasm, many participants referred to how the pandemic and associated policies illustrated the limits of solidaristic practices. While awareness of these limits was represented in all interview phases, in April 2020 they were mostly mentioned as exceptions. In October 2020, and even more profoundly in October 2021, people referred to unsolidaristic practices as a societal problem, particularly in the context of refusal to get vaccinated against COVID-19 or ignorance of protective measures, as this 70 + year old female participant illustrated:

*With regard to the anti-vaxxers, I really see this behaviour as a lack of solidarity and also as ignorant, yes. Or when people refuse to wear a mask or keep their distance, I think that’s also a lack of solidarity. […] They just think they won’t catch it. Or if they do catch it, they’re young and they think it won’t affect them badly. And yes, it’s also selfish, because they can also infect others. (participant 6, October 2021, see also supplementary quotes #22–23)*

Some participants described how differing opinions on how to handle the pandemic led to discussions and social exclusion within their social environment. For example, one childless female participant in her 30s reported that she felt like a “spoilsport” because she would avoid visiting her elderly parents even though other family members would:

*In my family, people don’t care in the sense that they say, ‘if grandma cannot see her grandchild, that’s so awful’. And I’m always a bit of a spoilsport. I’d reply, ‘well, I wouldn’t drive from [city 1] to [region in Germany] to visit my mother right now, even if I was very careful’. I would just have a really bad feeling if something were to happen. And then I’m a bit, yes, the spoilsport who overdoes it somehow. And this [role] is also quickly attributed to me. And on the one hand, I stand by my position, but on the other hand, some kind of opposing camps are forming, and I find that kind of unpleasant. (participant 15, October 2020)*

While these observations were rarely directly described as a demotivating factor to act in solidarity, they contributed to the weakening of the strong sense of togetherness that used to be present in the early stages of the pandemic. It supported societal polarization and the concentration of solidarity practices to those with similar viewpoints and behaviour.

#### Increasing costs

By October 2020, many participants described an increasing awareness of costs evoked by adherence to restrictions, such as personal limitations (e.g., refraining from seeing friends and family or not going abroad for vacation) and societal costs (e.g., economic regression). The motivation to act in solidarity for the benefit of society did not vanish but was described as increasingly costly to individuals and vulnerable groups as the pandemic progressed. For example, a young woman living alone in a rural area with a university education and long-term employment stated:

*Well, I had the feeling that a lot of people were really grateful when you brought them food, especially the elderly people in the retirement home who were super happy simply for seeing someone again. Of course, that was also a bit exhausting, because you thought to yourself, okay, I don’t have that much time […], [but] it was worth so much for the recipient and I think it was good that someone was there, whether it was me or someone else, I don’t think it would have mattered. (participant 33, October 2021)*

In October 2020 and particularly in October 2021, several participants described a feeling of fatigue caused by the constant and omnipresent state of emergency. Maintaining motivation to adhere to restrictions and social isolation was perceived as more costly than before. One self-employed mother of teenage children acknowledged the additional effort (and thus increased costs) of parents who could not bring their children to school or kindergarten as usual as an act of solidarity:

*And I also find it very solidaristic from the families of my kindergarten, […] that we somehow, [despite] our different attitudes, tried to protect each other in the pandemic. […] For many parents, this meant that their children had very little childcare and that they were really on their last legs. (participant 13, October 2021; see supplementary quotes #28–31 for other examples)*

On a societal level, economic costs and fears of financial uncertainty became more apparent as the pandemic progressed. For some participants, financial costs were eventually perceived as the limit of their solidarity, as this retired man with basic education who lived alone in a rural area described:

*We can no longer all sit at home and wait for the pandemic to somehow end, but we have to continue now because otherwise we will all go broke and be bankrupt and our jobs will no longer exist. But at some point, we had to get back to everyday life, yes, somehow, from the corresponding hardships, let’s say, purely financial hardships. And that’s where solidarity really ended. (participant 31, October 2021)*

In mentioning increased costs, participants directly or indirectly explained why they engaged more selectively in interpersonal solidarity practices.

#### Impairing incentives due to COVID-19 policy

Another potential reason for the waning enthusiasm identified by some participants was that COVID-19 policies impaired and disincentivized solidarity practices. For instance, a male participant in his 30s who lived with his spouse in an urban area held a university degree and long-term employment reflected on the internal contradiction of supporting others versus the social estrangement resulting from physical distancing:

*To be honest, I find it very difficult during the pandemic, when contact bans or restrictions are imposed, then I think it’s difficult to practice solidarity because it has something to do with interaction somewhere. I don’t think that telling people ’please stay home’ is really showing solidarity. People are told that this is somehow solidarity, but I think it is absolutely impossible. (participant 19, October 2021, see also supplementary quotes #32–41)*

Moreover, an unemployed man with a high school education who lived alone reflected on more widespread selfish behaviour due to the economic damages caused by the pandemic:

*In addition, all countries have been very badly affected economically and are now trying to get back on their feet. This means that egoism has been strongly encouraged by these things. And this solidarity, which is invoked again and again, is suffering and will continue to suffer in the future from this COVID-19 pandemic. (participant 3, October 2021, see also supplementary quote #33)*

Thus, the negative consequences of the policies evoked to protect people from getting infected were seen as a potential impairment to solidarity.

### Persistent importance of solidarity

Despite the waning enthusiasm and growing awareness about the costs and limits of solidarity practices that became apparent in many interviews, most participants still acknowledged the importance of solidarity in dealing with the pandemic. For example, this 30 + years old self-employed mother with a high school degree found that the crisis led to more practised solidarity:

*I think, one good thing about crises is that they make us very much focus on what’s really important. Because the other things simply have no place anymore. […] and [that], I think, also leads to more solidarity with each other. (participant 13, October 2021, see also supplementary quote #42)*

Moreover, several participants connected the challenges of the pandemic with future challenges by calling for inter-societal solidarity. For instance, in October 2021, participants emphasized the role of solidarity in overcoming crises like climate change or a major flood affecting three federal German states, in July 2021 (see supplementary quotes #43–46).

Further, most participants reported one or more examples of how solidarity served as a motivator for them to behave in a certain, pro-social way. One example was the efforts taken by participants to avoid infection and protect the vulnerable and the health care system.

*Actually, I have to admit that my main concern now is not to bring [the virus] to my family, for example, when I meet my elderly parents. Or when I somehow put other people at risk because I don’t know [that I’m infectious]. And maybe they don’t know that they are high-risk patients or have any pre-existing conditions or something. So simply not being a spreader, that’s the motivation, I think. (participant 13, October 2020, 30 + years old female with teenage children, self-employed with high school degree, living in an urban area, see also supplementary quotes #47–48)*

Other participants mentioned the same reasoning when considering getting vaccinated against COVID-19: Getting vaccinated or waiting in line for the COVID-19 vaccine was seen and felt as practised solidarity.

*And of course, solidarity also meant that all the priority groups, i.e. caregivers, nurses, doctors, nursing home residents, and so on, were vaccinated a few vaccine groups before the average person without a priority group. And all the young people who haven’t been vaccinated for a long time, all the students who haven’t been to university for a long time, even though they are certainly not the ones who get seriously ill most often, but/and who simply haven’t been to university locally for two years. (participant 49, October 2021, see also supplementary quotes #49–58)*

One woman in her 30s who lived alone, had long-term employment and higher education explained their persistent desire for solidarity with reciprocity, stating that she would help others because she would also like others to support her if she were in need.

*I have offered to help. So now with shopping, for example. <I: Would you do that again?> Yes. <I: What was the most enriching thing for you?> To support someone who needs help. Because if you were in that situation yourself […] you would be happy if you were in that situation if someone would take care of you. (participant 28, October 2021)*

Even though such direct references to expected reciprocity were rarely mentioned, the wish for some form of reciprocity became apparent in several other interviews. Yet, the main motivation people reported for engaging in solidaristic practices was the wish to contribute to their community. Reciprocity was more discussed in the context of a perceived unfair distribution of burdens (especially carried by the younger generations, caregivers, and small businesses), which we discuss in the following section.

### Unfair distribution of burden: calls for reciprocity and institutionalized solidarity

Another shift in people’s perceptions regarding solidarity practices was prompted by the realisation of how burdens evoked by the COVID-19 pandemic were unfairly distributed. In October 2020 and October 2021, many participants raised challenges regarding perceived inequalities, unfair treatment, and unilateral preference. Participants’ perceptions mainly focused on intergenerational reciprocity, caregivers, and economic burden. As a consequence of these observations, several participants pointed to insufficiently institutionalized forms of solidarity such as publicly funding support systems for those most affected by the pandemic.

#### Lack of reciprocity with particularly burdened social groups

In October 2020 and October 2021 interviews, participants increasingly stressed the negative impact of the COVID-19 pandemic on children, adolescents, young adults, parents, and professional caregivers. In recognising these vulnerabilities, they criticized the lack of social support for these burdened groups, which can be perceived as a lack of reciprocity: These groups were perceived as burdening high costs for acting in solidarity while being given little back in return.

##### Intergenerational reciprocity

For example, children and young adults who had shown consideration for older people and took on massive social costs were perceived to be abandoned by elderly people who were unwilling to get vaccinated:

*We were also very quick to show solidarity with the most vulnerable groups, the elderly. If they had not yet been vaccinated, we really made sure personally that we did not meet with the elderly on a large scale. Just to protect them. On the other hand, I really miss the solidarity with the younger people and the children and, of course, the schoolchildren. […] I would say there is a big gap to what the younger ones, as they say, have been deprived of to protect the older ones. And now the older people are not getting vaccinated and are putting the younger people at risk. I would say this is maximum non-solidarity. (participant 50, October 2021)*

Relatedly, several participants also expressed concerns about the social isolation of schoolchildren, long school closures and homeschooling.

*I see extreme problems in schools because the children now have virtually no serious symptoms and are coping very well with COVID. [And we as] adults have already found it difficult to reduce social contact. These are still, individuals who are still very much developing, and if you cut off their development, that’s a whole different ball game, and I see a huge problem there. (participant 2, October 2021 male, aged 46–60, living in a small town with children aged 12 and over, self-employed and university educated; see also supplementary quotes #59–63)*

Similarly, participants acknowledged the high costs the restrictions meant for adolescents and young adults, as this quote by an alone-living male participant in his 30s with higher education illustrates:

*I find it incredibly impressive how strictly adolescents adhere to [the restrictive COVID-19 policies], which is not a given as they have other needs at that age and usually react differently to rules. I’m impressed by how strictly they respect and adhere to them. (participant 4, October 2020, see additional quotes #64–69)*

These observations led some participants to call for more intergenerational reciprocity that should come in an institutionalized form (e.g., through additional policies). One mother of school children with long-term employment talked about “forgotten groups” and “lobby logic” (participant 9, October 2021) because children did not have a voice in the debate themselves. There were active calls for more solidarity towards these groups, such as from a young mother who worked at a university and lived in a large German city:

*But I somehow had the feeling that it’s the other way round, the lack of solidarity, these are young people who have really been deprived of a lot. […] And so many of them showed solidarity and really took a back seat. I found that really impressive. And to say again after a year, “Yo, I can’t do it anymore, I want to party now”, I can totally understand that. And to somehow pick on it and then somehow break it up with the police and so on, I sometimes thought, isn’t that perhaps actually lacking in solidarity? Because I think politics is already, I mean, there is such “agism”, it is mostly only discussed as older people are discriminated against. […] but I think in the pandemic it was rather the other way around. I think that somehow, especially the young people, a lot was demanded of them and very little was given back […]. (participant 32, October 2021)*

Another male participant in his 30s with higher education and long-term employment pointed to his helplessness in supporting younger generations and called for some kind of compensation:

*Just to take an example with young children. […] When you hear what paediatricians are saying about the increasing incidence of mental illness. I think you also have to create opportunities. Offer some kind of recreational activities to deal with this issue in some way. But also with the children, yes, just to give them a fun factor somewhere […]. Maybe, in the end, it’s not so dramatic, because people can somehow deal with stress and learn to deal with it, and these children, who knows, maybe in 20 years they’ll leave there as adults totally strengthened, because they, I don’t know, because they’ve already experienced something like this in their childhood, I don’t know. But right now I would say there is just a feeling of imbalance, but what you can do about it in detail, I don’t know. (participant 50, October 2021)*

##### Parents and caregivers

Parents of school or preschool kids were another group that some participants considered vulnerable from October 2020 on. Dealing with the social isolation of children, homeschooling and the uncertain situation with daycare places played a key role here, which posed major additional challenges for working parents in particular. This was drawn upon well in a quote by a woman in her 60s, working in the university context:

*Yes, the daycare centre was closed for a very long time and [my daughter] was already at the limit of her nerves because she had a small child who didn’t really have anyone to play with because they kept to themselves and they couldn’t invite anyone because they had just moved to the city. And things started to ease up when the [other] grandparents decided that they would stop staying away. They got over their fear of getting COVID, so to speak, because they realized that they [participants’ daughter and her partner] didn’t manage otherwise. And then they looked after [name of elder child] more often. (Participant 46, October 2020; see also supplementary quotes #70–71)*

In addition to parents, other caregivers, such as healthcare workers, kindergarten teachers, and childcare providers were identified by some participants as a vulnerable group. In the following quote, a kindergarten teacher in her 40s described her working situation in October 2021:

*And then having five little kids every day was a really big challenge and I was really scared a lot of the time…. I got scared when the kids got too close to me. I also noticed that I developed an aggressiveness that I had to suppress when the children had a snotty nose or sneezed. And in many situations, I’m always in the forest with the children, so I didn’t take care of the children indoors during that time. But we still eat together and it can happen that a child suddenly coughs. That’s when I almost went crazy inside. (participant 13, October 2021, see also supplementary quotes #72–75)*

#### Economic and global reciprocity

In addition to the above-mentioned social groups, participants also talked about insufficient reciprocity on national and international policy levels related to COVID-19. A young male participant living in a city and with long-term employment referred to a perceived imbalance between restrictions in the private and professional realms:

*What kept me thinking for a very long time was the issue that companies were not obligated to switch to home office, for example. The fact that there were so many restrictions at the private level, but not at the professional level, was connected to a lack of solidarity in my view. People had to forgo a lot in their private lives and went through with it, but as soon as it came to work, everyone had to go there anyway and was exposed [to a risk of infection]. (participant 34, October 2021)*

The lack of reciprocity and the resulting imbalance was also a matter of discussion when it came to state authorities. Several participants criticized the unfair distribution of restrictions and financial support, especially to the detriment of smaller businesses, such as hotels, restaurants, bars, theatres, or other cultural events:

*[…] Such measures [also] lead to […] poverty, because […] very often [certain] sectors were affected very unilaterally: In big companies, there were no restrictions, no obligation to work from home, but all restaurants had to close and all cultural events had to take a break. (Participant 34, October 2021, see supplementary quotes #76–80)*

On a global level, some participants talked about a lack of reciprocity regarding the global distribution of vaccines. For example, one young, alone-living woman from a rural area with long-term employment and higher education said:

*Yeah, at first I found it very shocking that the rich countries more or less grabbed the vaccine first and supplied themselves and then the poorer countries came along, I thought that was crazy because it was such a two-tier system. So if you didn’t have the money or you were late, you were out of luck. I didn’t think it was on the radar anymore that it was also about people. Somehow nobody wanted to know that there is a corona in Africa. I don’t only mean Africa, but other countries as well. I found this in India too. (participant 33, October 2021, see also supplementary quotes #80–84)*

In an attempt to promote better reciprocity, two young male participants suggested letting people unwilling to get vaccinated against COVID-19 carry any financial costs for health care caused by a COVID-19 infection themselves:

*What should be discussed in this context is that […] if you make a conscious decision not to be vaccinated, you also make a conscious decision to pay for the possible treatment costs yourself and not have them paid for by the community. (participant 39, October 2021, 30 + years old male living in urban area with a high school diploma and long-term employment, see also supplementary quote #85).*

## Discussion

In this qualitative study, we show how German residents’ perceptions of solidarity practices changed during the COVID-19 pandemic: In light of the acute crisis, participants emphasized their enthusiasm in April 2020 (as reported in previous studies) [14, 43] to manage the pandemic together as a society, to stick together, and to support each other. However, as the pandemic progressed, participants expressed a sense of fatigue and emphasized the high individual and social costs they bore to protect themselves and others. Solidarity practices increasingly focused on the immediate environment, i.e., family, friends, or immediate neighbours.

Our study complicates what others have framed as a “dereliction” of solidarity [44] or a decrease in interpersonal solidarity [45]: Despite waning enthusiasm, participants still acknowledged the crucial importance of solidarity practices to mitigate the negative consequences of the COVID-19 pandemic. They also extended the groups they considered to be vulnerable and, thus, in need of solidaristic support, which was also noted elsewhere [46, 47]. While in April 2020, solidarity and solidarity-motivated compliance with protective measures was often expressed to protect the health of elderly people, in the later stages, the social and economic burdens of other people (for example, children and teenagers, small businesses or the global south) were increasingly recognized. In line with our findings, the importance of intergenerational solidarity and the neglected support of the younger generation in Germany was acknowledged in a statement from the German Ethics Council, published in November 2022 [48]. As proposed in their statement, intergenerational reciprocity to help maintain solidarity practices in crises is best obtained through institutionalized solidarity. Our analysis draws a nuanced picture supporting this recommendation.

The observed persistent emphasis on the importance of solidarity for handling the COVID-19 pandemic despite waning enthusiasm to engage in solidarity practices might be related to growing awareness about the complexity of the societal problems caused by the pandemic: Participants reported a waning enthusiasm in fields where they did not perceive individual actions as helpful anymore. Instead, they called for institutionalized forms of solidarity, such as support systems for healthcare professionals, disadvantaged school children, and people who come under financial pressure due to the pandemic restrictions. These were perceived as more sustainable and efficient to support those in need than individual solidarity. Yet, policymakers in Germany repeatedly called for solidarity on an individual level and asked people to “show responsibility for each other” [49, 50]. Our findings could also be understood as a reaction against such politicization of solidarity as a concept instead of practising it by implementing institutionalized forms of solidarity and reciprocity to mitigate perceived and existing inequalities. Consequently, the reported waning enthusiasm for solidarity can be understood as a reaction to a perceived lack of institutionalized reciprocity and injustices.

### Implications for practice

Sustained solidarity practices and compliant individuals are key to maximising public health benefits in times of a pandemic [6] but also to managing global crises more generally. Our findings illustrate the importance of perceived reciprocity on the institutional level. Thus, for people to be motivated to practice solidarity and to engage in pro-social behaviour, policymakers should pay special attention to how different parts of the public perceive reciprocity in times of crisis. If possible, they should implement measures to foster reciprocity when calling for solidarity, for example through social welfare policies and an active appreciation of those carrying particularly high costs. Interestingly, our findings indicate that these social welfare measures do not have to be targeted toward everybody but rather towards those who are perceived as particularly burdened: in the case of COVID-19, for instance, elderly people, families with small children, young adults, healthcare professionals, or those who are self-employed.

Furthermore, it is important to provide concrete recommendations for what acting in solidarity means and entails. Our participants’ statements evoked the sense of being alone in figuring out how to put political appeals to solidarity into practice. Finally, financial support for different groups in the crisis should be communicated transparently and visibly to the general population through a government that acts on a reciprocal, solidarity-based role model. Reciprocity only supports solidarity if people recognize it.

### Strengths and limitations

Drawing from a rich, unique data source collected during the pandemic as part of the SolPan Research Commons, this qualitative study complicates the analysis of solidarity practices during COVID-19. It amends existing quantitative inquiries with a more nuanced understanding of people’s perceptions of interpersonal, group-level, and institutionalized solidarity. While this analysis focused on German data to get a more in-depth, context-specific and longitudinal understanding of people’s perceptions of solidarity, other SolPan publications focus on a more international assessment: A study including data from April 2020 and October 2020 from nine countries, for instance, reveals that the wish for more institutionalized solidarity is a finding that is relevant beyond the German context [38]. In addition, compliance with social distancing, mask-wearing, and COVID-19 vaccination was shown to be motivated by solidarity to some degree [6, 18, 47, 51–54].

Still, we acknowledge this study’s limitations: First, the sample of interviewed participants is not representative of the German population. Even though we aimed to maximize variety in perspectives by controlling for demographic factors (see Table 1), people from disadvantaged socio-economic backgrounds and in precarious living conditions (e.g., refugees, homeless people) are underrepresented and people with higher education and long-term employment are overrepresented. Since individuals in such privileged life conditions are more in the position to carry costs related to solidarity practices, this overrepresentation likely influenced the findings of this study. Indeed, the majority of illustrative quotes come from participants with higher education. Also, we had no participants who self-identified as COVID-denier or anti-vaxxer in our sample. This needs to be considered when interpreting the findings of this study and indicates that future research should especially focus on the lived experiences and views of disadvantaged populations.

Because we wanted to limit the timeframe of interviews for each panel to capture experiences during the same moments of the pandemic, theoretical saturation could only be assessed post hoc during data analysis. We applied a pragmatic approach to theoretical saturation as proposed by Jacqueline Low [41] by presenting reflections on what aspects might be unsaturated from our dataset. However, due to the described study design, no additional recruitment took place in the case of missing perspectives, which is a limitation: For example, most of our participants trusted the government and were largely supportive of their interventions. Also, as the interviews were held either via telephone or video calls, the interviewers were limited in interpreting non-verbal gestures and, depending on the connection, in rare cases problems of understanding, which were cleared up by repeated questioning.

Data collection and analysis are situated in Germany and people’s perceptions were strongly situated in the local setting and influenced by pandemic experiences and policies as well as the epidemiological development of the pandemic in the country. Germany represents a Western country in continental Europe, where the concept of solidarity has a strong anchoring in politics and bioethical research traditions as compared to the Anglo-Saxon world [55, 56]. As such, solidarity is strongly anchored in the culture, traditions, and policies in Germany, which likely influenced people’s reporting. Still, solidarity has been reported to become more prominent in public and scholarly discourse beyond continental Europe in light of COVID-19 and other international crises [55, 57, 58], rendering the findings relevant to these contexts as well.

## Conclusions

The COVID-19 pandemic brought with it many challenges, especially those related to sustained solidarity practices. This study provides longitudinal empirical evidence from Germany supporting the notion that solidarity practices did not vanish or deteriorate over time. Instead, we found a persistent desire to maintain solidarity practices. Nevertheless, the pandemic came with various challenges to follow this desire: From the broadening of who was considered vulnerable, to the dwindling enthusiasm and simultaneous growing fatigue and social as well as mental costs and the perceived gaps in the reciprocity of solidarity practices. The perceived lack of reciprocity at both intergenerational and institutional levels while the perceived costs of acting in solidarity increased are apparent in this study. Yet, solidarity practices can only be maintained if the costs for the individuals and society are bearable.

Thus, this study bears implications for public health policy: Policymakers should assess how people perceive reciprocity in times of crisis and should implement measures to foster reciprocity when calling upon solidarity. However, we hold that this is only possible if social support systems based on solidarity are not limited to, but built, maintained and strengthened beyond any politically defined crisis. Such kind of support networks contribute to forming a society whose members maintain solidarity also during times of crisis.

### Electronic supplementary material

Below is the link to the electronic supplementary material.

Supplementary Material 1: SolPan interview guides (Team Germany)

Supplementary Material 2: Supplementary quotes

## Data Availability

To protect the anonymity of participants, full interview transcripts are not publicly available. Further data excerpts for supporting the results can be retrieved from the authors upon reasonable request (contact medizinethik.med@tum.de).

## Abbreviations

COVID-19: Coronavirus Disease 2019
SARS-CoV-2: SARS Coronavirus 2
SolPan: Solidarity in Times of a Pandemic
