# Supplementary material for: Solidarity and reciprocity during the COVID-19 pandemic: a longitudinal qualitative interview study from Germany
Source: BMC Public Health. 2024 Jan 2;24:23. doi: 10.1186/s12889-023-17521-7 (PMC10763370; doi:10.1186/s12889-023-17521-7)
Supplement: Supplementary file 1 — Supplementary Material 1: SolPan interview guides (Team Germany) [file 12889_2023_17521_MOESM1_ESM.docx]

| **Context** |  | **Quote** |
| --- | --- | --- |
| **Waning enthusiasm and perceived limits of solidarity practices** | | |
| Examples of solidarity practices at the beginning of the pandemic | 1 | Yes, although / Yes, there is a neighbourhood help service in our district that is organized online. There is also the possibility of ordering things from individual shops, which you can either pick up at the door or have delivered to your home. For example, our bookstore, which we all support. That's how it works. And when we need something, we call them and you can either pick it up at the door on a Saturday. Or during the week they bring it by bike and deliver it to the door. So things like that are done here. (participant 1, April 2020, 60+ years old male with long-term employment and higher education, living as a couple in a rural area) |
| Examples of solidarity practices at the beginning of the pandemic | 2 | So we are participating as well. And some of my friends have already done it. Especially when it comes to buying these coupons or this [name of support group] that makes these offers e.g., to go shopping, yeah. (participant 17, April 2020 30+ years old female living with children under 12 in urban area, long-term employment, higher education) |
| Examples of solidarity practices at the beginning of the pandemic | 3 | There are shopping helpers, students who help with gardening, students who help with picking strawberries, and so on. I think these things are great. I don't know how many **people volunteer.** Then we have relatively well-functioning **church congregations that provide helplines**, that provide delivery services. For example, we can just ask someone to do the shopping for us or something like that. (participant 47, April 2020, 60+ years old retired female living as couple in rural area, higher education) |
| Examples of solidarity practices at the beginning of the pandemic | 4 | Yes, that's what you sign up for. And it also says that you can put something in the mailbox of the person who put their name on it. Or just ring the doorbell. And then I thought, yeah, why not. So if someone puts something in my mailbox, why shouldn't I? I have a lot of time now. (participant 22, April 2020, 18+ years old female living alone in urban area) |
| Examples of solidarity practices at the beginning of the pandemic | 5 | And yes, **I do see people showing solidarity**. We also put up notes in the hallway and said: Hey, we'll go shopping for you if you need any help, these people and neighbours. (participant 32, April 2020, 30+ years old female, living with spouse in urban area, precarious employment, higher education) |
| Waning enthousiasm | 6 | I mean, at the beginning of the pandemic, I had, it was a little bit like the influx of refugees that we used to have. […] there was only six months of a welcoming culture and you almost had the impression that we were now a really nice society and then it turned into the complete opposite. And now it was the **same, in this initial phase of the pandemic you already had the feeling of a very strong sense of togetherness**. Towards the end of the pandemic, however, you could already see that everyone was discussing things from a very strong point of view, and that **envy effects** were emerging. But 20 percent of the population has not yet been vaccinated. If you continue to try to squeeze them out by making vaccination and testing incredibly expensive and completely excluding them from everything, then **that doesn't unite a society, it divides it**. (participant 2, October 2021, 45+ years old male living with adult children in small town, self-employed, higher education) |
| Waning enthousiasm | 7 | I think at the beginning of the pandemic, I saw a lot of that, where people were buying things for each other, I don't know. <I: 2020> And I think **that went so much down the drain. So I'm very negative**. So for me as a collective behaviour I found, I have such a negative, such a pale (mouth) [after] taste now in the end. And I have to say that I'm no better personally. So I don't think I've behaved any more [in solidarity] than anybody else. (participant 42, October 2021, 30+ years old female living with children under 12 in an urban area, long-term employment, higher education) |
| Waning enthousiasm | 8 | Well, in principle, the question is what this [the pandemic] will bring us. At the moment, I don't know whether there is more solidarity or whether a **counter-development** is starting to develop. [...] that people are taking even more care of themselves and looking even less at others. (participant 43, October 2020, 30+ years old male living with children under 12, small town, self-employed, higher education) |
| Waning enthousiasm | 9 | [...] When there are acute problems, I've learned that somehow you have to stand together first. **But the longer the problem exists and the longer the problem lasts, the more normal the situation becomes again.** (participant 34, 18+ years old man living alone in urban area, high school degree, long-term employment) |
| Waning enthousiasm | 10 | **I think that the big phase of growing together was last year.** And that this year people just kind of pedalled or just continued what they built last year. (participant 11, 30+ years old female living with spouse in urban area, long-term employment, higher education) |
| Opposing view on waning enthusiasm | 11 | I have the impression that **solidarity has not diminished in the last year**. At the beginning, I thought you saw more people without masks or something, yes? People who perhaps rebelled against it a bit or didn't realize it. I think that's gotten better somewhere, a bit more solidarity. (participant 16, October 2021, 60+ years old male, retired, living in rural area, higher education) |
| Vanishing initiatives | 12 | Yes, it was mentioned. But I couldn't say much about it because I hadn't noticed much. But yeah, I mean now in my house the neighbours had put up notices that they could help with shopping and so on. But I think it's more like **it's receded and the notice is no longer there.** But maybe people organized themselves online somehow, I really don't know now because **I'm not that active**. (participant 37, October 2020, 18+ years old female with high school diploma, living alone in an urban area) |
| Vanishing initiatives | 13 | Initiatives, what other initiatives are there? I don't think I can answer that question. The one that I thought I would get involved in or show something, **I didn't get any feedback and then I kind of left it alone**. (participant 28, October 2020, 30+ female living alone in small town, long-term employment, higher education) |
| Vanishing initiatives | 14 | B: So you mean like shopping for neighbours and so on? I: For example, exactly. B: Yeah, well, in our area now / **They're a little bit so worn out, I don't even know how to describe it** / So there's not really anything going on anymore. I don't see much activity. (participant 42, October 2020, 30+ years old female with children under 12, living in urban area, long-term employment, higher education) |
| Vanishing initiatives | 15 | I didn't really notice much of it because everything was loosened again. No, really. **That went away relatively quickly**, including that portal where you could help each other, offer help, I think I mentioned that at the time, that wasn't the case anymore. (participant 17, October 2020, 30+ years old female with children under 12, living in urban area, long-term employment, higher education) |
| Vanishing initiatives | 16 | Initiatives? Nope, well, I once got in touch with some kind of, what's it called? Shopping help for older people or people who need a bit of support. **They didn't get back to me at all**. So I didn't get any feedback**. So there doesn't seem to be any demand**. At least here in my region, probably. Initiatives, what other initiatives are there? I don't think I can answer that question. The one I thought I would get involved in, I didn't get any feedback and then I kind of left it alone. (participant 28, October 2020, 30+ female living alone in small town, long-term employment, higher education) |
| Vanishing initiatives | 17 | **So we didn't take up the offer from our daughter, who lives nearby, to have her do the shopping for us. We did that ourselves and we want to keep doing it**. I also think that here in our region, most, the vast majority of people are sensible enough to follow the hygiene rules. And that's why I think it's relatively safe. (participant 45, October 2020, 70+ years old retired male living with spouse in rural area, higher education) |
| Vanishing initiatives | 18 | I can't think of any organization/ The DRK [German Red Cross] was completely occupied with maintaining the test and vaccination centres and at least in our region, in the rural area. And apart from that, apart from the medical doctors and the clinics, there were, in my impression, relatively **few private or association-based initiatives that would have especially promoted the well-being of the population**. (participant 45, October 2021, 70+ years old retired male living with spouse in rural area, higher education) |
| Vanishing initiatives | 19 | I have to dig really deep here. Yeah, so neighbourly help in the house, for example. We live next to a lady who is not good on her feet somehow and, I think, a bit anxious about COVID and I've asked her a few times if I should bring her something. But I think she was afraid of that too and refused. And a friend of mine in the house, he often bought things for an elderly lady. (participant 21, 18+ years old female living alone in urban area, long-term employment, high school diploma) |
| Still existing initiatives | 20 | So my daughter**, she still went out voluntarily and helped** old people go shopping and drove them to the doctor. I say, 'Watch out, I'll tell you', she did that even before the vaccination. And she says, 'Mom, I have a face mask on and the other woman or man also has a face mask on' and so on. I say, 'Well, you have to sort it out for yourself' and so on. 'If you want to do it, then do it" and toi, toi, toi, it went well. In the end, I was also pleased that she did it, yes, because that way she just did something good. (participant 23, October 2021, 60+ years old retired woman living with spouse in small town, high school diploma) |
| Still existing initiatives | 21 | So **I think they still exist,** at least there was a leaflet in our letterbox recently. But as we didn't make use of it ourselves, I didn't really follow it up. But I think that **if you need help, you'll get it**. So I haven't read that it no longer exists. I think it's still active, especially for older people or single people or people who are unsure and don't know what to do. Especially when it comes to shopping, I think that still exists here, yes. (participant 43, October 2020, 30+ years old male living with children under 12, small town, self-employed, higher education) |

| **Limits of solidarity and societal polarization** | | |
| --- | --- | --- |
| Noncompliance | 22 | But the few times I've been to the pharmacy or a coffee shop, **I think it's a shame how often people have their masks hanging under their noses or don't have any masks on at all**. Especially in a cafe, when making coffee, I think it sucks. Yeah, I don't know. But on the other hand, if I think about it on a meta level: **I can also understand that there are signs of fatigue, like that.** I notice that myself, I also have the feeling that I can't isolate myself here for 1.5 years with the little baby. But yeah, it's a small price to pay for a lot of security, so I don't understand why people don't do it (participant 32, October 2020, mother of a newborn child living in urban area, higher education) |
| Compliance | 23 | B: Yeah, I get annoyed sometimes when people don't really follow through. But now I know, at least in my circle of friends, that everyone is really vaccinated and then I don't have such a problem with it. It's always a question of solidarity and community. (participant 28, October 2021, 30+ female living alone in small town, long-term employment, higher education) |
| Individual interest vs public interest | 24 | Yes, so I would say politicians who didn't make clear statements because they assumed that it would have a negative impact on the election results. I think that's a total lack of solidarity. (participant 42, October 2021, 30+ years old female with children under 12, living in urban area, long-term employment, higher education) |
| Suffering global solidarity | 25 | In addition, all countries have been hit very hard **economically** and are trying to get back on their feet. This means that **selfishness has been very strongly encouraged** by these things. And this solidarity that is constantly being invoked is suffering and will continue to suffer from this Covid-19 pandemic. (participant 12, October 2021, 70+ years old male living with spouse in urban area, higher education) |
| Limits of solidarity at the workplace | 26 | But then I asked the two ladies, who also have children of their own, and said that I was very disappointed that this was just / I mean, it would have been a huge relief. If I'd known there was special leave / Every time I thought I was going to work from home with the kids and everything, I took a regular day off because I thought I couldn't make it. And well, as it turned out, I was / I thought, of course, they just have to discuss it and that's it. And then this special leave was extended to the whole institute. And announced. But nothing happened. And then it just turned out that one lady said she wanted to talk about it again, and they apparently discussed it for half an hour because the bosses said, "Well, you can work from home," and they were worried that if it was made public that there was such a special leave, people would just take advantage of it. (participant 42, October 2020, 30+ years old female with children under 12, living in urban area, long-term employment, higher education) |
| Limits of solidarity | 27 | In terms of solidarity, I think the general mood has, as I said, simply become a little calmer since the vaccine came out. And when society becomes calmer, then ultimately it is usually more supportive of its fellow human beings, that's my impression. (participant 20, 30+ years old male, living with children under 12 in rural area, long-term employment, high school diploma) |
| **Increasing costs** | | |
| Growing perception of personal costs | 28 | And also where everyone is really anxious to hug the other person. But they don't. (participant 24, October 2020, 45+ years old female living with spouse in urban area, long-term employment, higher education) |
| Growing perception of personal costs | 29 | What I think is particularly important is that there are no large gatherings of people, that's just true, I think that, I assume that if there are large gatherings of, I don't know, 150, 200 people upwards, then the danger is simply that the virus can spread quickly, that the hotspot is created, so to speak. And it's just important that you keep doing this, even if it's difficult. **Personally, I find it difficult. As a young person who likes to party, but you just have to.** You can do it for another six or nine months until a vaccine is on the market and in circulation. (participant 30, October 2020, young male with high school diploma, living in a rural area with long-term employment) |
| Growing perception of personal costs | 30 | Once the family, so I have two adolescent or just grown-up granddaughters, and out of fear of catching the virus, we only saw each other rarely for high school graduation, graduation, and celebrations of any kind. My brother turned [60+], **he couldn't celebrate** that, so it's really thinned out. (participant 18, October 2020, 60+ years old retired woman. living alone in urban area, higher education) |
| Growing perception of personal costs | 31 | Well, I felt like a lot of people were really grateful when you brought them food, especially the older people in the nursing home were really happy because they just got to see somebody again. Of course, it was also a little bit stressful because they thought, okay, I don't have that much time and now I'm being talked to so much and I'm actually the person who's actually getting all the fears and worries and I don't really know him at all, I just keep/yes, just older gentlemen in the neighbourhood. [...] I would definitely do it again because I think you have to stick together and help each other, especially in a pandemic like this. **And even though I was a little annoyed at times that I was being talked to for an hour, it was worth so much to the person** and I think it was also good that somebody was there, whether it was me or somebody else, I don't think it would have mattered. (participant 33, October 2021, 18+ years old female living alone in rural area, long-term employment, higher education) |
| **Impairing incentives through COVID-19 policy** | | |
| Impairing incentives | 32 | Exactly, she was a little bit better now after the lockdown, during the lockdown I tried to help her from a distance, which was also a little bit difficult, of course, because you can't do more than try to call, but if the person is in there / that they don't answer the phone or something **and you're not allowed to enter the apartment, according to the rules, of course it's difficult to help the person**. [...] Exactly, if the person doesn't answer the phone or something, there's no other way to help them. (participant 27, October 2020, 18+ years old male student living in an urban area, basic education) |
| Impairing incentives | 33 | I get the feeling that people have to learn again to live together and not just care about themselves. I find on the one hand the pandemic triggered people to care about others, helping elderly people, for example, and on the other hand, it has created a distance where everybody went their own way. (participant 33, October 2021, 18+ years old female living alone in rural area, long-term employment, higher education) |
| Impairing incentives | 34 | Yes, I'm just thinking about my mother or my siblings. It's really stressful when you can't visit them the way you actually want to. Or holding them in your arms. The fact that you keep your distance, that you realize that people find it difficult not to get too close to you. [...] With my mother, I solved it by taking her in my arms anyway, but then with my head to one side so that we didn't breathe on each other. (participant 36, October 2020, 60+ years old retired male living with spouse in rural area, higher education) |
| Weighing between social isolation and protection against infection | 35 | We were able to meet up with our family, they live in the same house. But they have schoolchildren, and it was of course very difficult for the working parents and for us, who are often looking after the children. Dealing with the precautionary measures, when **the parents tightened the precautionary measures on their own initiative to protect us. That really hurt**; how should you explain to a three-year-old that he can't go to grandma's today. (participant 31, October 2021, 70+ years old retired man, basic education, living in rural area) |
| Weighing between social isolation and protection against infection | 36 | We are back in contact with my other two daughters. We've seen each other relatively often. **And the grandchildren too. And that was a huge joy for us, of course.** Because this abstinence for 3 months was terrible. And we also met up with friends again. The summer allowed it. So we had coffee together, dinner together, outside. But we actually limited ourselves more compared to what other people were doing, with distances and not indoors if we could avoid it. **The only exception we make is actually with family.** If they've announced a visit and it's raining, then we're also inside with them. (participant 47, October 2020, 60+ years old retired female living with spouse in rural area, higher education) |
| Weighing between social isolation and protection against infection | 37 | **The biggest challenge is meeting with the parents**, with my mother-in-law, because it's always with mixed feelings: I hope we don't put her at risk. [...] We weigh it up a little bit. Yeah, well, it's very difficult. Because of the lockdown, I haven't seen my mother-in-law for a long time. She lives alone, my father-in-law died and I don't know, I think it was just before the summer holidays, I spoke to her on the phone and then I realized on the phone that she was on the verge of a breakdown, that she was in a very, very bad state, which I hadn't really noticed before. And I said, "It's no use. We have to go now, because who knows if we're going to see her**. And yes, we are weighing it a little bit, how important is social life for you? And how big is the risk of the coronavirus?** (participant 14, October 2020, unemployed female living with children under 12 in small town) |
| Weighing between social isolation and protection against infection | 38 | Yes, I have a [90+] year old mother [...] who I used to visit every 3-4 weeks. I didn't do that until July, which was of course relatively hard for the old lady. [...] In July, we were on vacation for two weeks, which was like a quarantine, like not working as a doctor for two weeks, and then we spent a week with my mother. **And now that the numbers were so good, we've been back more often.** But less so now. [...] And it's the same with my parents-in-law, who are also very old, and we actually went out for dinner once a week. We had cancelled that for a long time, but started again when the numbers were so good in the summer. [...] I think we'll continue doing that, because they've suffered so much from the isolation that they're probably rejecting it now. [...] It's their wish that we see each other more often. (participant 2, October 2021, 45+ years old male living with adult children in small town, self-employed, higher education) |
| Weighing between social isolation and protection against infection | 39 | And of course, it's always an issue. Who can come to visit, who can't come to visit? Because the numbers are going up again. My sister and my father both work in the healthcare system, as doctors, and then you always ask yourself: "Hm, is it a great idea for them to come and visit or not"? At the same time, of course, I don't want to deprive them of your grandson. But yes, you do have to **consider the benefits and risks when arranging visits.** And sometimes that's just a real shame. that this is not as carefree as it would be if it weren't for the pandemic. (participant 32, October 2020, mother of a newborn child living in urban area) |
| Weighing between social isolation and protection against infection | 40 | So I was considerate of my mother because I realized that she was, well, more anxious or cautious. I respect that. [...]But other parts of the family, my godmother, uncles, cousins and so on: we've had several family gatherings over the course of the year, just a week ago. And **we try not to let ourselves be restricted in that respect**. In any case, these people are over 70. (participant 19, October 2020, 30+ years old male living with souse in urban area, long-term employment, higher education) |
| Weighing between social isolation and protection against infection | 41 | My grandmother, for example, lives alone, and because of the pandemic, she simply had a lot less social contact, because now I, as her granddaughter, and also the other grandchildren and family members did not come to visit as often. On the one hand, **they were afraid of infecting her** or maybe catching [COVID] on the way to my grandmother's. **So older people like my grandmother might be a little, yes, lonely**. (participant 37, October 2021, 18+ years old female with high school diploma, living alone in an urban area) |
| **Remaining importance of solidarity** | | |
| Crisis forsters solidarity practices | 42 | I don't think the pandemic has changed the way we live together at all, but I think the pandemic has shone a light where it hurts. I think that people who were alone during the pandemic were alone before. And the people who were overburdened before the pandemic, who are now somehow connected to families or something, yes, they were overburdened before. And maybe they were overburdened, but the pandemic just turned the tables. So maybe it was just a little bit too much. And the people who were flirting with conspiracy theories before, the people who are flirting with conspiracy theories today, were flirting with conspiracy theories before. But the pandemic has tightened the screws a little bit more. And I think that maybe it intensified certain processes of alienation. And at the same time processes of solidarity elsewhere, but I also think it's a very - so maybe it's just my hope, I don't know, but I've experienced both **somehow. That people come a little bit closer together because they somehow realize, okay, I can't do this alone,** I have to ask. So I have to build a network and then it works somehow, yeah. (participant 32, October 2021, 30+ years old young mother living in urban area, short-term employment, higher education) |
| Role of solidarity in other crises | 43 | Well, I thought **the solidarity that was shown during the floods was incredible**, that so many people drove up to help. And that employers paid for it anyway and said, 'yes, it's totally okay that you're driving up, it's good that you're helping, you'll still get your salary'. (participant 33, October 2021, 18+ years old female living alone in rural area, long-term employment, higher education) |
| Role of solidarity in other crises | 44 | And pure solidarity is [...] what we experienced during the **flood disaster** this year. I mean, in one of the richest countries in the world, between 150 and 200 people died in two days due to heavy rainfall, and an unprecedented volume of donations was mobilized within a very short space of time. I don't think so much money has ever been raised for anything in Germany in such a short space of time, I think that's really great. And the fact that it had to be publicly announced: 'Please refrain from driving further into these areas, there are enough helpers. We can help at the moment/ More helpers are not needed,' which actually shows how great solidarity can be. (participant 19, October 2021, 30+ years old male living with souse in urban area, long-term employment, higher education) |
| Role of solidarity in other crises | 45 | Yes, I actually observed, experienced and practiced solidarity myself recently at **climate protests**, when we stood together in a group of climate activists against [exhibition in German city]. And we were also exposed to police truncheons and pepper sprays and stuff, so we experienced violence, police violence. And that was very solidaristic in a way because the violence was spread over many heads and many faces [...]. And it would have been easy for individuals to leave and expose the others to even more violence. And I think it's quite a lot of solidarity to stay there anyway and take the beating together and then support each other emotionally afterwards [...] (participant 13, October 2021, 30+ years old female living with teenage children in urban area, self-employed, high school diploma) |
| Role of solidarity in other crises | 46 | So when the floods came, in the Rhineland and on the Moselle and so on, we had a **donation marathon**. So I cooked meatballs, potato salad, then posted it on Instagram and then the money that was raised was donated [...] and it was really well received. [...] And yes, for me that also means solidarity, simply seeing that there are people who have lost everything and I'm sitting here on my warm couch like this. That makes me a little, well, thoughtful. (participant 24, October 2021, 45+ years old female living with spouse in urban area, long-term employment, higher education) |
| Protecting the elderly | 47 | I mean, we personally are not so afraid of infection because we are all healthy and are convinced that we would overcome this disease [COVID-19] well. But I mean, of course you don't necessarily want to have it and, yes, **you do abstain from things to protect others who might be more at risk**. I also have a colleague in the office who is over 60 and, yes, I mean, I don't necessarily want to infect her. So you also think about other people who are simply more at risk, that's a motive, too. (participant 5, October 2020, 30+ years old female with teenage children, living in rural area, long-term employment, higher education) |
| Protecting the elderly | 48 | And yes, I took it very seriously at the time, so for older people and for risk groups, I saw it more as something like the curfew or all the other restrictive measures, that I should keep to them as much as possible because it was just so that I could be a carrier. **Not because I was worried about myself and I didn't really panic. But because of other people**, I say, just to take responsibility. (participant 22, April 2020, young female participant who lived alone in an urban area) |
| Vaccination as act of solidarity | 49 | So of course we all practiced solidarity ourselves, because we just **accepted the vaccination sequence**. Solidarity is a very big issue, that's clear. (participant 50, October 2021, 30+ years old young father living in a small town, long-term employment, higher education) |
| Vaccination as act of solidarity | 50 | For example, the fact that **my colleague was vaccinated after I asked her explicitly that it was very important to me that she was vaccinated when I was pregnant**. Because otherwise I don't know how we can work together without me having to keep a very large distance from her, and that would be at the expense of our lunch together, because I just say that I wouldn't do it otherwise. I think that's an act of solidarity on her part and I give her a lot of credit for that. I think she did it because I said it was so important to me. (participant 49, October 2021, 18+ years old female living alone in urban area, short-term employment, higher education) |
| Vaccination as act of solidarity | 51 | And I've gotten a flu shot at the urging of my doctor, and I'll get one again this fall. Simply because the arguments make sense to me. Because people like me - I'm in my mid-sixties now - could very well end up in the hospital if they catch the flu. If I get pneumonia or something like that. And **I definitely don't want to be blocking hospital beds** if there's a risk of a pandemic. First of all because of myself, but also because of all the people who might need to go there. And that has changed my attitude. (participant 47, October 2021, 60+ years old retired female living with spouse in rural area, higher education) |
| Vaccination as act of solidarity | 52 | Yes, I actually got a flu shot in 2020 for the first time in my life. Especially for me, it wasn't even about getting the flu myself, **it was just about taking the pressure off the hospitals**. I was supposed to get the vaccine this year, but somehow, I missed my appointment. I'm a long-term person, and I'm convinced that natural infections improve and provide long-term vaccine protection. I think this is actually supported by the current data for COVID. So I think a single vaccination along with a natural infection is the best vaccine protection or the best protection, and I think it's similar with influenza. (Participant 42, October 2021, highly educated young mother aged 30+ with long-term employment living in the city) |
| Vaccination as act of solidarity | 53 | Yes, the vaccination itself, I had massive side effects after the second vaccination. You often hear that young people in particular have that. So I was really completely out of action for another 24 hours. As I said, amongst other reasons, **I did the vaccination because then I wouldn't have to feel guilty anymore**. I did [feel guilty] before, when I decided to meet up with friends somewhere and celebrate together even though we weren't allowed to. We did it anyway in winter. But then we always had a bit of a guilty conscience when we were with our grandparents and hoped that we wouldn't infect them. And [the vaccination] made it easier, I didn't have to worry about this anymore and I also had a bit of security for myself that I probably wouldn't be the one infecting elderly people. (participant 30, October 2021, young male with high school diploma, living in a rural area with long-term employment) |
| Vaccination as act of solidarity | 54 | For me, solidarity meant that some groups have taken a back seat for other groups during the pandemic. My group, for example, **the 40-plus group, whose turn [to get vaccinated] came relatively late**. But even more so the young people, who were of course hit particularly hard. The people who were 15 or 16, teenagers, and then up to 20-plus students and so on, who are usually very mobile, who are just starting to really explore the world, and they were actually completely locked in, just like the old people. But the elderly were the first to receive the vaccine, so to speak, and that was a form of social solidarity. (participant 26, October 2021, 40+ years old male living with children under 12 in urban area, self-employed, higher education) |
| Vaccination as act of solidarity | 55 | [...] it was always very clear to me that I would have my children, my teenage children vaccinated as soon as possible, simply **to protect the grandmothers**, to put it bluntly. So that was also an important topic in our family, yes. (participant 13, October 2021, 30+ years old female living with teenage children in urban area, self-employed, high school diploma) |
| Vaccination as act of solidarity | 56 | Well, of course, it's probably also an act of solidarity that I got vaccinated or that many people say they don't really feel like it, but okay. It's just, it's not about that, it's about the big picture and the more and the quicker people get vaccinated, the sooner we can all get back to normality. Or, yes, that we clearly stick to the rules to protect others. (participant 21, 18+ years old female living alone in urban area, long-term employment, high school diploma) |
| Vaccination as act of solidarity | 57 | So of course, we all practiced solidarity when we simply accepted the vaccination prioritization. Solidarity is a very big issue, that's clear. We also practiced solidarity with the vulnerable groups, i.e. the elderly. In other words, when they hadn't yet been vaccinated, we really made sure - me personally - that we didn't meet with elderly people. Simply to protect them. (participant 50, October 2021, 30+ years old young father living in a small town, long-term employment, higher education) |
| Vaccine prioritization | 58 | So in the beginning it was a fight about the vaccine, and at first I thought it was a fair discussion about criteria, and then we looked at who actually fell under those criteria. And then at some point it was no longer about criteria, it was about 'we want it too'. And so **I just found some groups lacking in solidarity in the way they suddenly conducted the debate.** And I thought that this had nothing to do with the political regulations, but only with the way in which such a political discourse is conducted in public. (participant 9, October 2021, 30+ years old female living with children under 12 in small town, long-term contract and higher education) |
| **Intergenerational reciprocity** | | |
| Vulnerability of school children | 59 | The biggest challenge? [… ] Reminding the kids [...] that things are different now, and dealing with the **disappointment** when bigger parties don't happen, including the kids. That **you can't just have a kid's birthday party** and invite 15 people. (participant 26, October 2020, father of schoolchildren living in an urban area) |
| Vulnerability of school children | 60 | So **we told our two kids that one of their best buddies could still visit us,** so they wouldn't be completely lonely during their vacation here. But maybe that wasn't quite the rules. But it was really just one, not like a group and always the same one. (participant 2, October 2021, 45+ years old male living with adult children in small town, self-employed, higher education) |
| Vulnerability of school children | 61 | I think a school lockdown is bad. In principle, a much better element here would be to create corona-free zones via tests, where it is relatively safe and corona-free and more or less almost normal teaching. And where there are coronavirus infections, you could create more or fewer zones and regulate access via zones. [...] There are also parents who don't have the internet. (participant 36, October 2020, 60+ years old female living alone in small town, long-term employment, high school diploma) |
| Vulnerability of school children | 62 | So the area that is closest to me from my previous job, of course, is that children, yes, that **education is given priority**. That there is really no consideration at all of a second lockdown as far as schools are concerned, but instead it is asked: What do we need to do to minimize the risk in schools and daycare centres? And then actually do it. (participant 18, October 2020, 60+ years old retired female living alone in urban area, higher education) |
| Vulnerability of school children | 63 | Conversely, **I really miss the solidarity towards the younger ones and thus the children and of course also the school children.** I don't have a schoolchild, but I have schoolchildren in my family. And I would say that there is a great discrepancy between that and what the younger ones, as they say, have been deprived of to protect the older ones. And now older people are not getting vaccinated and are putting younger people at risk. I would say that this is maximum non-solidarity. (participant 49, October 2021) |
| Vulnerability of adolescents and young adults | 64 | And I would say that there is a lot of work to be done. Because you can see quite clearly which age groups have the appropriate status in our society and who has a mouthpiece, so to speak, and who does not. And yes, it has to be said that young people, and I don't just mean small children, but also **young people who are somehow sitting in schools or lecture halls or even at home, have really lost out.** In terms of quality of life, in terms of time. Especially now, these wild young years, when you should be living a very, very cool life. Nobody gives them that year and a half back, or I don't know how much longer. And you have to say that you really have to make sure that it's cushioned in some way or that there's some kind of - I don't know if there is such a thing - but **some kind of compensation**. So something has to be given back to them, because otherwise I don't think it's balanced at the moment. (participant 50, October 2021, 30+ years old father of a toddler with higher education and long-term employment, living in a small town) |
| Vulnerability of adolescents and young adults | 65 | **But when young people stand together and amuse themselves, I realize it's not cool**. And probably if someone of the same age came and wore a mask, they'd laugh at them. I think that´s just peer pressure. I mean, I still remember what I was like when I was young. I did a lot of things that I wouldn't have done if I'd been alone. And maybe you have to accept that to a certain extent? **Include the young people** and say, yes, they also have a right to this kind of social life, to hang out in cliques and party together. (participant 18, 60+ years old retired woman. living alone in urban area, higher education) |
| Vulnerability of adolescents and young adults | 66 | But what is still an issue for me at the moment is that we talk a lot about these forgotten groups. So the students, the **students as forgotten groups**, and that's where I'm concerned in terms of solidarity or, well, maybe it's not solidarity at all. But solidarity is often directed towards the other group and the fact that people are now looking at forgotten groups, that everything is always fragmented into groups. (participant 9, October 2021, mother of a schoolkid with long-term contract and higher education, living in a small town) |
| Vulnerability of adolescents and young adults | 67 | [T]here is a young orchestra member who is a student and [...] found all these restrictions incredibly bad. Of course, because they hadn't been vaccinated for a long time and had huge problems with studying and working. [...] At some point he had enough. He replied very sharply to an e-mail of an elderly pensioner and said that as a young person he felt he was being taken for a ride by someone who lives in his own house with a high pension and great security and he could have been vaccinated for [...] many months [while] he's practically living on the breadline, he's studying under terrible conditions and as a young person he has to be considerate of the elderly and [...] he feels he's being treated very badly [in return]. Chapeau, I thought it was great that he raised his voice. (participant 47, October 2021, 60+ years old retired female living with spouse in rural area, higher education) |
| Vulnerability of adolescents and young adults | 68 | All the young people, they haven't been vaccinated for a long time, all the students who haven't been to university for ages. Although they’re certainly not the ones who are most likely to get seriously ill but […] now just haven't been to the university physically for two years. (participant 50, October 2021, 30+ years old young father living in a small town, long-term employment, higher education) |
| Opposite viewpoint of young adults | 69 | So there were no initiatives where people said: "Oh, we have to help the children." I mean, they're all adults. They're [20+], right? You don't have to help them much anymore. So we didn't really help them much for COVID reasons or anything. I mean, I tried to organize a vaccination appointment for our eldest son somehow, which is self-evident and what we usually do. (participant 16, October 2021, 60+ years old male, retired, living in rural area, higher education) |
| **Caregivers** | | |
| Vulnerability of young parents | 70 | So I think that one of the biggest and most emotionally stressful things was that there was not always a harmony between what was said and what actually happened. Especially in the day care centres of course / there are much stricter hygiene regulations. For one [older daughter] this means that / **We had a month where she was sent home at least one day a week because she had a runny nose and so on**. And that's what the employer sees / The employer sees: "Everything is normal and back to normal". But it's not. And it's not a question of whether you can deal with it or not, it's just this emotional feeling of being alone, which I think is very difficult for us, or for me anyway. (Participant 42, October 2020, highly educated young mother with long-term employment living in the city) |
| Vulnerability of young parents | 71 | Especially for those who tend to be alone and also for those who live in very close quarters where emotions or aggression can arise quickly.[...] I think it's really emotionally stressful when living with kids and partner in a 60-square-foot apartment with four people and they can't go outside at all. And then it's fall or winter and you can't even go to the playground because it's wet and rainy. So I imagine that's bad. (participant 8, October 2020, 45+ years old male living with spouse in urban area, long-term employment, higher education) |
| Daycare personnel | 72 | I can perhaps mention a case from our daycare centre. The rules there are very confusing, and I think that the nursery staff [...] are essentially overworked. So they have a lot of hygiene measures that they have to carry out now. And they also have the usual number of children and of course, I can understand that / They are the interface to many children and are potentially exposed to many illnesses. They are sometimes very nervous. (Participant 42, October 2020, highly educated young mother with long-term employment living in the city) |
| Supporting health care personnel | 73 | Almost all parties are now saying the same thing in the federal election campaign, for example, that people who work in the healthcare system should be better paid and that the nursing profession should be made more attractive. Bluntly, that clapping is not enough. And that's all correct, yes, I completely agree. I hope that it happens, let's put it that way. So I think the awareness is here. I also hope that it can be implemented satisfactorily. And perhaps there is also a rising awareness, that's what I hope, that a hospital does not exist to make profit but to, well, help the sick and contribute to society in solidarity, so to speak. (participant 29, October 2021, 30+ years old male living with spouse in urban area, long-term employment, higher education) |
| Supporting health care personnel | 74 | And [the policymakers] should also go there and say, I can't let staff overwork for a longer period of time. They may be able to do 100 or 150 percent for a while, but at some point [the system] breaks down. And that's just the way it is and the burden on people is there, too. That means they need a break. In other words, we have to look after and nurture this staff. (participant 36, October 2021, 60+ years old female living alone in small town, long-term employment, high school diploma) |
| Supporting health care personnel | 75 | For example, the nursing sector, which has been taking to the streets in [city 2] for four weeks now and protesting for better working conditions. And I mean, a year ago we were all clapping from the balconies for the nursing staff. It's a bit closer to us now than it was before, which perhaps has something to do with the pandemic. We've perhaps also understood a little more that we can't just, well, if we just try to privatize everything like this, we might not benefit from it in the end. (participant 13, October 2021, 40+ years old female living with teenage children in an urban area, self-employed, high-school diploma) |
| **Economic burden** | | |
| Unfair distribution of burden (economically) | 76 | in [city 2] **three shops have closed**. So two restaurants, and yes, it's just a chain, but I don't feel so sorry for them, but **I do feel sorry** for the restaurant owners. They just didn't survive, yeah. So I feel very sorry for them, yeah. (participant 46, October 2021, 60+ years old female living alone in small town, long-term employment, high school diploma) |
| Unfair distribution of burden (economically) | 77 | B: Yeah, I mean, there are measures like **closure of premises and so on, some of which have been unfairly distributed**. We are seeing now that a lot of businesses, small businesses like pubs, hotels, restaurants, bars or theaters, music venues, are really going down the drain. And I would have liked to see more differentiated decisions based on good data. So the closure of the Philharmonic, the theater, until recently, I don't know. But then to somehow make sports events possible for 20,000 people, that was somehow too much. (participant 18, October 2021, 60+ years old retired female living alone in urban area, higher education) |
| Unfair distribution of burden (economically) | 78 | Yes, I do find it difficult that cultural events are not being allowed more broadly. I feel that there are **double standards**. Soccer events are no problem, if 60,000 people are somehow there in the stadium, I don't see a major problem. But on the way to the stadium, you know, I've been to a lot of soccer matches, what it's like on public transport when 60,000 people are being transported back and forth [...]. Or you see on TV, even now at the mourning ceremony for the Ahr victims with the Federal President and so on, and that was also packed in a huge hall, everyone sitting close together without masks. Yes, I think it's very stressful when you see it like that and then you hear that some children in schools still have to wear masks and I don't think that's the same level. So we're in such an imbalance at the moment. (participant 2, October 2021, 45+ years old male living with adult children in small town, self-employed, higher education) |
| Unfair distribution of burden (economically) | 79 | Yes, I'm going to say no to larger gatherings. This coming weekend I'm also going to pass on the first, the first opportunity to go to the [soccer club in city1] soccer game. But then there's also the fact that **I don't really understand why soccer**, the first and second Bundesliga**, is given so much more attention than other events, including cultural events**. We've also cut back on cultural events, but we'll take part in them if they have a hygiene concept. (participant 10, October 2020, 60+ years old male living with spouse in small town, long-term employment, higher education) |
| Unfair distribution of burden (global) | 80 | I still think it's a real shame that in Africa, for example, people say, well, we can send down the vaccine that we don't need, and so few people can be vaccinated. **I think it's a real shame that people don't get together to make sure that everybody gets vaccinated**. [...] And then also, for example, that **so many shops had to close down because they couldn't survive**. I think that's also very bad, that livelihoods have been threatened and destroyed. (participant 46, October 2021, 60+ years old female living alone in small town, long-term employment, high school diploma) |
| Unfair distribution of burden (global) | 81 | But it's big business and doing business with poor countries is not a profitable business**. So the fact that in some countries only two percent are vaccinated, even in some European countries only 20, 30 percent, it's really bitter**. And we don't know how we're going to be hit again by variants, whatever. It's an enormously complex issue, but it's not the only issue where the so-called Western countries, which is most of Europe and North America and Australia, the rich industrialized countries, are doing well at the expense of other countries. This is not the case with vaccination, but it has always been the case. Yes, even in health policy, people only think about HIV/AIDS in the same way. (participant 18, October 2021, 60+ years old retired female living alone in urban area, higher education) |
| Unfair distribution of burden (global) | 82 | [The rich industrialized countries] simply have more influence, more money and so on to buy things. In this respect, it is of course **unfair in terms of global politics and geopolitics**. [...] in this case, a certain **egoism of the rich countries** takes hold. Unfortunately, that is the case. And at least they have now started to pass on the vaccine quantities that would eventually go to waste and are now also helping poorer countries to procure vaccines. And I'm also pretty sure that the prices for the vaccines / they are now / that is / demand in the rich countries has become relatively low and therefore I believe that the overall price of the vaccines is also decreasing so that the poorer countries can also afford them. (participant 45, October 2021, 70+ years old retired male living with spouse in rural area, higher education) |
| Institutionalized forms of solidarity practices | 83 | And yet there were a lot of organizations that went down [to other countries] and helped. I think that's valuable too, it's great that they did that. (participant 33, October 2021, 18+ year old woman living alone in a rural area, long-term employment, higher education) |
| Institutionalized forms of solidarity practices | 84 | And apart from that, I think there's also solidarity in the discussion about how to distribute vaccines to countries that don't have the means to buy them themselves. So I think in principle it feels very solidary to me that there is this discussion and that there are some voices that say this is important. (participant 13, October 2021, 40+ years old female living with teenage children in an urban area, self-employed, high-school diploma) |
| Take on self-responsibility | 85 | So for me, when you say you don't want to be vaccinated at all, it's very difficult because then I have very little understanding if you have something that makes you say: "Yes, okay, I need help and I need this and this and this." Then I would say: "Okay, **you had the responsibility to get vaccinated, to protect yourself from this. Now you have it. And now you want, now you demand help?"** Yes, we're still a welfare state, so you still have to help, so I see it a little bit differently when people get sick. (participant 27, October 2021, 18+ years old male student living in an urban area, basic education) |
