# Supplementary material for: Solidarity and reciprocity during the COVID-19 pandemic: a longitudinal qualitative interview study from Germany
Source: BMC Public Health. 2024 Jan 2;24:23. doi: 10.1186/s12889-023-17521-7 (PMC10763370; doi:10.1186/s12889-023-17521-7)
Supplement: Supplementary file 2 — Supplementary Material 2: Supplementary quotes [file 12889_2023_17521_MOESM2_ESM.docx]

**Supplementary File – Interview-Guides**

The interview guides were designed by a joint effort of the SolPan Research Commons. These interviews were preceded by an initial round of interviews held in April 2020 (T1). This interview guide is published here:

Consortium, SolPan, Interview Guide 'Solidarity in Times of a Pandemic' (April 11, 2021). Available at SSRN: <https://ssrn.com/abstract=3824361>

**Interview Guide October 2020 (T2)**

**1. How have you been since the last time we spoke?**

1. If you think back to the last time we spoke, in April - what has changed since then? *(follow up: in your work life, personal life)*
2. What has been most challenging for you (your family etc.) since we last spoke?

[*possible follow-up: if the respondent has not raised this, for example in the answer about what has changed, ask about their use of technologies in the practices and views described; if respondent mentions children, ask them about school arrangements. Also use this first section to follow up on specific topics/situations discussed in the first interview.*]

**2. How did you experience the measures evoked to contain viral spread?**

1. What do you do to protect yourself and others? Why? What motivates you? *(Examples: getting ill, passing the virus on, legal or financial penalties)*
2. Is there anything you voluntarily refrain from doing, even if it is allowed *(Examples: meeting people, wearing face masks even if it is not compulsory). [Follow up with Why?]*
3. Can you give me an example of a time when you made an exception to the rules? What happened? How did you feel?
4. What do you think of the reactions of the general public regarding the pandemic? Can you give an example?
5. What are your experiences with other people’s reactions and behaviours since we last spoke? (what struck you in the way people reacted and behaved to the easing of the restrictions in the summer?)
6. Can you think of any initiatives that came up during the COVID-19 pandemic to support people?

**3. What are your views on how the government has handled the crisis since we last spoke?**

1. On reflection, what do you think about the measures implemented in the first phase of the pandemic? What do you think of school reopenings? *[Follow ups: Can you please elaborate; Why etc.]*
2. What government measures did you find particularly important? Do you still follow them? *[Follow up: Why? How?]*
3. Do you think that any people are taking the recommended restrictions too lightly, or on the other hand, any people are taking restrictions too seriously or even obsessively? Can you provide some examples? How do you explain this behavior?

*[be attentive to whether interviewees talk about hard governmental measures or recommendations/advice - try to get a differentiated picture - if possible]*

**4. Where do you get information/news about the pandemic?**

1. Which sources do you trust and why?
2. Have the sources you trust changed since the last time we spoke? If so, how and why?

*[If respondents have not raised these topics in the answers to the other questions, please ask about the following topics (please feel free to adjust the wording to fit your specific country context)]:*

**A. VACCINATION**

1. There has been much talk of vaccines being developed to prevent infection with the virus. What are your views on these hopes for a vaccine? How and where do you follow these developments? [follow up: and why these particular info sources?]
2. If there was a vaccine for Covid-19, what would be reasons/conditions for you to get it/not get it? And how would you go about getting it?
3. Who should get vaccinated first if there were scarce resources? Why? [Getting behind motivations of acting - whether others should take it but not oneself]
4. Does a potential COVID-19 vaccine compare differently for you than other decisions you have made regarding a vaccination (either yourself or other family members)- for the annual flu, for example?
5. Who do you trust most in communicating about this, and why?

**B. FACE MASKS**

1. Do you wear a face mask? Why (not)?
2. What are your experiences with face masks?

**C. TRACKING AND TRACING**

1. As you may know, track-and-trace systems are in place [explain if necessary]. Have you heard about it? Do you have experience with this system?
2. Has the way you use technologies (e.g., Skype, social media, etc.) changed since the pandemic?
3. What are your thoughts about contact tracing? Did you make any personal experience with it

**5. What are your thoughts about the upcoming weeks and months?**

1. What are your expectations of the upcoming weeks and months? (and your work/family life)?

**Is there anything that you would like to add?**

**Thank you for participating!**

**Interview Guide October 2021 (T3)**

1. **General views about and experiences in the pandemic**
2. One year living with the COVID-19 pandemic has passed since we last spoke. What have been the greatest challenges for you and those close to you? *(let talk first – then if appropriate, specify differences between personal experiences during 2020 and 2021) (if appropriate, specify: working life, personal life, social life)*
3. Are there people who have experienced particular *pandemic-related challenges* near you? F
   (if yes) Are you aware of any *initiatives or actions* (by people, organisations, etc) *to help* them?
   (if participants report that they themselves have supported others): What has *your experience been with providing support* in this manner? Would you do it again? What was the most rewarding? What was difficult about it?
4. **Compliance with and contestation of policy restrictions**
5. What government measures were/are easy to follow? What was/is difficult? W
   hat are the benefits and the problems of each one of these measures?
6. What is your experience with the EU Digital COVID Certificate? What are the benefits? What are your concerns? Have you discussed this with others? What topics were discussed?
7. How do you feel about the behavior of other people with regard rules and measures?
8. How did your behavior change because of the pandemic? E.g., regarding protective measures you follow even though you are not legally obliged to?
9. **Vaccines**
10. What are your personal experiences with Covid-19 vaccinations? *(if necessary, specify: trust/concerns/fears; definition of priorities; particular controversies towards specific brands; hopes/uncertainties related to “immunity”; distribution of vaccines among countries)*
11. What do you think has gone well and wrong in the vaccination process in your country? (if necessary, specify government/ministry of health actions, planification, communication)
12. Can you think of any circumstances where mandatory vaccination would be indicated/reasonable?
13. *[Adapt to whether participant has stated previously whether and how they have been vaccinated]* What has changed/or do you expect to change in your life after you got/get vaccinated?
14. How has your perception about vaccines (Covid and other) changed through the pandemic?
15. **Public debates**
16. How did you experience the media coverage during the pandemic? Through which media?
17. To what extent did the media coverage influence your views and behavior regarding protective and restrictive measures? Do you have a specific example? *[Follow up: How did you perceive e.g. wearing a mask, vaccination, economic support measures?]*
18. **Future**
19. How do you expect the pandemic will change your personal life in the next 12 months?
20. Is there anything that you have done/were doing before the pandemic that you would no longer be willing to do?
    *[follow up: why, why not, what has changed? If you think about the role of work in your life, and in particular, any jobs that you have held in the last months and years, how has this role of work changed through the pandemic?]*
21. Do you know someone who is no longer willing to do something (work related or not) that they were doing before the pandemic? Can you give some examples?
22. How has your sensitivity towards your own health changed with respect to pre-pandemic times? *(potential follow up question: are you more concerned or less concerned about your health than before?) Why? What about other people?*
23. How do you expect/anticipate the pandemic will change how we organize living together on the same planet?
24. What lessons do you think the governments/politicians should learn from the pandemic?
25. What needs to happen in your opinion for the pandemic to be really, completely over?
26. **Solidarity**
27. What does solidarity mean to you?
28. Maybe you could share a concrete example where you experienced or witnessed solidaristic behavior?
29. Maybe you could also share your experience with non-solidaristic behavior? *(ask for an example of solidaristic and non-solidaristic behavior each)*

We have asked all our questions but if there is a topic which is particularly important to you or that we have missed to address, this would be a good time to mention it.

What are your impressions on this interview (and the interviews we did in the past)? Why did you decide to take part in this study?

**Thank you for participating!**
